# What Are the Risk Factors for Malnutrition in Older-Aged Institutionalized Adults?

**DOI:** 10.3390/nu12092857

**Published:** 2020-09-18

**Authors:** Lorenzo M. Donini, Blossom C. M. Stephan, Aldo Rosano, Alessio Molfino, Eleonora Poggiogalle, Andrea Lenzi, Mario Siervo, Maurizio Muscaritoli

**Affiliations:** 1 Department of Experimental Medicine—Medical Pathophysiology, Food Science and Endocrinology Section, Sapienza University, 00185 Rome, Italy; lorenzomaria.donini@uniroma1.it (L.M.D.); Eleonora.poggiogalle@uniroma1.it (E.P.); Andrea.lenzi@uniroma1.it (A.L.); 2 Institute of mental Health, Division of psychiatry and Psychology, School of Medicine, Nottingham University, Nottingham NG7 2QL, UK; blossom.stephan@newcastle.ac.uk; 3 Agenzia Nazionale per i Servizi Sanitari Regionali, 00100 Rome, Italy; rosano244@gmail.com; 4 Department of Translational and Precision Medicine, Sapienza University, 00185 Rome, Italy; alessio.molfino@uniroma1.it; 5 School of Life Sciences, Queen’s Medical Centre, The University of Nottingham Medical School, Nottingham NG/ 2QL, UK; Mario.Siervo@nottingham.ac.uk

**Keywords:** malnutrition, elderly, nutritional status

## Abstract

Malnutrition is common in older adults and is associated with functional impairment, reduced quality of life, and increased morbidity and mortality. The aim of this study was to explore the association between health (including depression), physical functioning, disability and cognitive decline, and risk of malnutrition. Participants were recruited from nursing homes in Italy and completed a detailed multidimensional geriatric evaluation. All the data analyses were completed using Stata Version 15.1. The study included 246 participants with an age range of 50 to 102 (80.4 ± 10.5). The sample was characterised by a high degree of cognitive and functional impairment, disability, and poor health and nutritional status (according to Mini Nutritional Assessment (MNA), 38.2% were at risk for malnutrition and 19.5% were malnourished). Using a stepwise linear regression model, age (B = −0.043, SE = 0.016, *p* = 0.010), depression (B = −0.133, SE = 0.052, *p* = 0.011), disability (B = 0.517, SE = 0.068, *p* < 0.001), and physical performance (B = −0.191, SE = 0.095, *p* = 0.045) remained significantly associated with the malnutrition risk in the final model (adjusted R-squared = 0.298). The logistic regression model incorporating age, depression, disability, and physical performance was found to have high discriminative accuracy (AUC = 0.747; 95%CI: 0.686 to 0.808) for predicting the risk of malnutrition. The results of the study confirm the need to assess nutritional status and to investigate the presence of risk factors associated with malnutrition in order to achieve effective prevention and plan a better intervention strategy.

## 1. Introduction

Malnutrition describes a deficiency of a wide range of nutrients and results in significant adverse effects on body composition, function, and clinical outcome. It is common in older adults, particularly in nursing home residents where prevalence is estimated to be between 20 and 90% due to different diagnostic criteria [1,2]. Anorexia and reduced food intake are mainly due to age-related changes in physiological, pathological, and environmental factors. Moreover, increased energy and nutrient requirements, comorbidities, polypharmacy, as well as psychological and socio-economic status may negatively influence energy balance, promoting the onset of malnutrition [3,4,5].

Malnutrition is associated with functional impairment, reduced quality of life, and increased morbidity and mortality [6,7,8]. The incidence of complications in malnourished nursing home residents is 27% compared to 16% in well-nourished subjects, while mortality is three-fold higher (12.4 versus 4.7%, respectively) [9]. Malnutrition may worsen age-related sarcopenia (in turn increasing the risk of disability), decrease immune response, and precipitate the onset of pressure sores [10,11,12]. On the other hand, cognitive decline and depression are negatively influenced by malnutrition and may act as a maintenance factor for poor nutritional status, determining changes in feeding behaviour (especially loss of interest in food) [13], thus starting a vicious cycle. All these conditions may affect health care costs because of longer hospital stays, delays in recovery from diseases and disability [14], and an increased rate of hospital readmissions [15].

Identifying risk factors for malnutrition is of utmost importance to better guide intervention and prevention strategies. In all care settings, it is important to characterize the determinants of malnutrition, as intervention strategies may need to be more general and focused on the improvement of structural characteristics of the hospital/nursing home facility as well as on the specific rehabilitation process of the individual patient. Although several studies have been conducted in Italy on the prevalence of malnutrition in nursing homes [16,17], few data are available concerning risk factors for poor nutritional status in older institutionalized adults.

Although the main risk factors for malnutrition in nursing homes are known, the prevalence of malnutrition remains high. It is therefore important to continue to emphasize this contradiction between research and clinical activity in order to contribute to the improvement of care for nursing home residents.

The aim of this study was to explore the association between health (including depression), physical functioning, disability and cognitive decline, and risk of malnutrition in an older Italian population of institutionalized adults.

## 2. Methods

Participants were recruited from seven nursing homes in the Latium region, Italy. Full details of the methodology have been previously published [18]. In brief, inclusion criteria included (1) age > 50 years and (2) resident in the facility for a minimum of six months. Patients were excluded if (1) they required artificial nutrition; (2) their height and weight could not be assessed. All patients who met inclusion/exclusion criteria were considered eligible for the study.

The study protocol was approved by the Local Ethics Committee-Sapienza University, Policlinico Umberto I of Rome, Italy (protocol number 847/17). Oral and written informed consent was obtained from all participants or their legal representatives where individual consent was not possible (e.g., due to severe cognitive impairment).

All participants completed a detailed multidimensional geriatric evaluation administered by a trained investigator. The assessment lasted approximately two hours.

Disease-related multi-morbidity was assessed using the Cumulative Illness Rating Scale (CIRS) [19] that rates malignancies in 14 areas (cardiac, vascular, haematological, respiratory, EENT (eye, ear, nose, throat, larynx), upper gastrointestinal (GI), lower GI, hepatic and pancreatic, renal, genitourinary, musculoskeletal, neurological, endocrine-metabolic, psychiatric/behavioural) on a five-point scale: 1 = no problem affecting that system; 2 = current mild problem or past significant problem; 3 = moderate disability or morbidity and/or requires first-line therapy; 4 = severe problem and/or constant and significant disability and/or hard-to-control chronic problems; 5 = extremely severe problem and/or immediate treatment required and/or organ failure and/or severe functional impairment. From the results, two scores were created: (1) severity index (average of the scores of the first 13 categories, excluding the “psychiatric/behavioural pathologies”); (2) comorbidity index (number of categories in which a score ≥3 was obtained, excluding “psychiatric/behavioural pathologies”).

Global cognitive function was assessed using the Mini Mental State Examination (MMSE) test [20]. This includes questions based on five domains: orientation, registration, attention and calculation, recall, and language. Scores range from 0 to 30 with higher scores indicating better cognitive function. Using standardized cut-offs, MMSE scores were categorized into two groups: impaired/possible dementia (≤18) vs. not-impaired (≥19).

Impairments in Activities of Daily Living (ADL) were assessed using the Katz [21] scale. This assesses disability in six areas: bathing, dressing, ability to use the toilet, mobility, continence, and feeding. Each area was rated on a three-point scale: 0 = does not require help (no difficulty); 1 = some difficulty (may require assistance); 2 = severe difficulty (requires assistance). Scores for each area were combined (score range: 0 to 12), with higher scores indicating greater disability.

The Short Physical Performance Battery (SPPB) [22] was used to assess lower extremity function concerning three domains: gait (walking speed), balance (standing balance), and strength (repeated chair rises). Each domain contributes 0 to 4 points. Scores across the three domains are added (total score range: 0 to 12), with higher scores indicating higher levels of functioning. The SPPB score was kept continuous for the analyses.

Depression was assessed using the Geriatric Depression Scale Short Version (GDS) [23], a 15-item test with higher scores indicating an increased probability of depression. A score greater than five typically signals the need for further investigation. GDS scores were kept continuous for this analysis.

### 2.1. Anthropometric Measurements

Height, weight, and calf and mid-arm circumference were measured. All methods followed the procedures as described in the “Anthropometric standardization reference manual” [24]. The same tools were used in all facilities: A SECA scale 86 (200 kg, to an accuracy of 100 g, certified/homologated as class III), a flexible metallic tape (200 cm; accuracy: 1 mm), and a telescopic stadiometer (200 cm; accuracy: 1 mm).

### 2.2. Modified Mini Nutritional Assessment (M-MNA)

Nutritional status was assessed using the Mini Nutritional Assessment (MNA) [25]. The MNA includes 18 items: (1) medical assessment (*n* = 8 items: mobility, psychiatric, and neurological problems including stress, prescription medication use, living arrangements, functioning, mode of feeding); (2) anthropometric assessment (*n* = 4 items: body mass index (BMI), weight loss during the last three months, and mid-arm and calf circumference); (3) dietary assessment (*n* = 4 items: number of daily meals and daily consumption of protein, fruit and vegetables, and fluids); (4) subjective assessment (*n* = 2 items: self-view of nutritional status, self-reported health status). A weighted score is assigned to each item. The total score ranges from 0 to 30 (>23.5 points: normal, adequate state of nutrition; 17–23.5 points: increased risk of malnutrition; <17 points: malnutrition).

For the main analyses, the score was modified (M-MNA) to remove subjective items related to nutritional and health status (*n* = 2 items), psychiatric status (*n* = 2 items assessing psychological stress/acute disease and presence of dementia/depression), mobility (*n* = 1 item assessing ability to move about or being bed/chair bound), and comorbidity (*n* = 2 items; one relating to medication use and one relating to the presence of pressure sores or skin ulcers). This minimizes recall bias (in older participants with cognitive impairment/dementia) and redundancy with cognitive measures [26]. The total score ranged from 0 to 18.5, with higher scores indicating better nutritional status. New threshold values were used based on the methods by Snalders et al. [26] using percentage equivalent scores for the original cut-off scores as follows: ≤10 = malnourished, 10.5–14 = at risk for malnutrition, and >14 = normal nutritional status. Based on this grouping, a binary variable was created: normal status vs. malnourished (at-risk and poor nutritional status groups combined).

### 2.3. Statistical Analyses

Continuous variables are presented as means and standard deviation (SD), or as median and interquartile range (IQR) for non-normally distributed variables. A percentage (number) was used for categorical variables. Gender differences in nutritional status and demographic, health, functional, cognitive, and depression variables were tested using the chi-squared test for categorical variables or the t-test (or Mann–Whitney U test for non-normally distributed variables). All analyses were completed using Stata Version 15.1.

### 2.4. Prediction of Malnutrition

Multivariable linear regression analyses, controlling for age and sex, were used to test the association(s) between nutritional status (M-MNA, continuous) and each independent variable (cognition, disability, comorbidity, severity, physical performance, and depression), separately. Significant variables were then entered into a stepwise linear regression analysis (both forward and backward stepwise tested, *p* = 0.05) to determine independent predictors of nutritional status. The magnitude of associations was reported as beta coefficients and standard errors. Logistic regression analysis was used to determine how well the stepwise selected model predicted nutritional status. Predictive accuracy was tested using the area under the curve (AUC) with 95% confidence intervals (95%CI). The Hosmer–Lemeshow test was used to assess model goodness of fit.

## 3. Results

In total, 254 participants (66.9% female) were recruited; 4 subjects were excluded due to inclusion/exclusion criteria, while 4 subjects denied consent to participate (response rate 98.4%). The MNA score was therefore calculated in 246 participants. Age ranged from 50 to 102 (mean = 80.4, SD = 10.5). The percent of missing data was low and ranged from 0% (for age, sex, disability/ADLs, SPPB) to <1% (for the MMSE, GDS, and CIRS). Therefore, a complete case analysis was used.

The clinical and functional characteristics of the present cohort are shown in Table 1. In particular, the sample was characterised by a high degree of cognitive and functional impairment, disability, and poor nutritional and health status. According to MNA, 38.2% of the patients were at risk for malnutrition and 19.5% were malnourished. No patients were receiving oral nutritional supplements. When considering the gender, females were found to be significantly older, had significantly reduced physical performance, and had significantly greater impairment in ADLs compared to males (see Table 1).

As shown in Table 2, when controlling for age and sex, all variables except health status (severity and comorbidity indices) were significantly (and positively) associated with the M-MNA total score. When all variables were entered into the stepwise linear regression model, only age (B = −0.043, SE = 0.016, *p* = 0.010), depression (B = −0.133, SE = 0.052, *p* = 0.011), disability (B = 0.517, SE = 0.068, *p* < 0.001), and physical performance (B = −0.191, SE = 0.095, *p* = 0.045) remained significantly associated with the M-MNA score and were therefore retained in the final model (adjusted R-squared = 0.298)**.**

The logistic regression model including age, depression, disability, and physical performance was found to have high discriminative accuracy (AUC = 0.747; 95%CI: 0.686 to 0.808, *n* = 245) for predicting the binary outcome of normal nutritional status vs. impaired (at-risk or malnourished). The model was well calibrated, as indicated by the H-L test (chi-squared = 240.09, *p* = 0.468).

## 4. Discussion

In the present study, we found that age and physical and mental health were all significant predictors of poor nutritional status. These results may have important implications in the definition of risk factors for the development of malnutrition in older adults.

As previously described, the prevalence of malnutrition, as determined by the reduced-MNA test, was high in our cohort (19.5% of females and 14.6% of males were classified as malnourished; 38.2% of women and 37.8% of men were at risk of malnutrition) [18]. This is in agreement with previous studies indicating a prevalence of malnutrition in older adults admitted to nursing homes ranging from 37% to 82% [27]. However, it is important to note that the variability in prevalence rates likely is due to the different nutritional assessment used across the studies concerning differences in how malnutrition is defined (e.g., type of assessment, whether continuous or categorical scores are used) as well as sample characteristics (e.g., age, disease-related comorbidity, living arrangements). Nevertheless, given the high prevalence of malnutrition, the results highlight the need to monitor nutritional status in older residents. In fact, a nutritional risk screening and a comprehensive nutritional assessment are the basis for the control and reduced risk of malnutrition/disease-related comorbidity, as well as for the nutritional diagnosis and nutritional interventions adapted to patient needs [25]. As stated in the ESPEN guideline on clinical nutrition and hydration in geriatrics, “the process of nutritional care for older persons consists of several steps which are based on systematic screening for malnutrition. If there are any indicators of nutritional risk, a detailed assessment should follow to substantiate the diagnosis of malnutrition and as a basis for the definition of individual treatment goals and the development of a comprehensive nutritional care plan” [28].

Malnutrition was associated with increased age and high levels of disability and depression similarly to results from other studies. With regard to age, previous studies have found that the prevalence of protein-energy malnutrition increases with age, particularly in individuals 70 years old [3,8,9]. In fact, the ageing process causes a decrease in the function of various organs (e.g., digestive and cardiovascular systems, muscle), thus affecting eating behaviour, nutrient absorption and metabolism and, finally, the balance between nutritional needs and intake [10,11,12,13]. With regard to depression, Smoliner et al. [29] found that depression, assessed using the GDS, was the only independent risk factor for malnutrition in a multiple regression analysis, whereas age, sex, care level, number of prescriptions, and self-caring capacity were not significant. However, the authors suggest caution in interpreting these results. In fact, the relationship between depression and malnutrition is complex, and it remains unclear whether depression is the cause or the consequence of impaired nutritional status. With regard to physical function, the results are complex. Within a nursing home setting where meals are prepared, and therefore it would be expected that a person would have adequate access to food regardless of their physical status (e.g., compared to free-living individuals), disability may increase malnutrition risk via a number of pathways: (1) concomitant conditions; (2) treatment side-effects for a comorbid disease which, alone or in combination, could be related to disability; or (3) an overall reduction in food intake due to feeding impairments. 

In contrast, while cognitive status, physical performance, and disease-related comorbidity were significantly associated with malnutrition in the univariable analysis, they were dropped from the multivariate analysis. This is in agreement with some, but not other studies. Indeed, a study by Kaipainen et al. [30] found that when defining nutritional status using a binary variable, excessive polypharmacy, lower MMSE score, and a higher GDS-15 score were all independently associated with malnutrition or risk of malnutrition.

### Limitations

In our study, lack of significant findings could be due to multicollinearity. For example, physical performance is correlated with disability, while age and depression may stand for the impact of cognitive impairment and comorbidity on the nutritional status. Further, in a cross-sectional study, reverse causality is likely (depression, dementia, and physical impairment cause poor nutrition, but in turn they can be promoted by undernutrition). The study is cross-sectional; therefore, it is not possible to test for causation or investigate changes in nutritional status over time.

The age range listed in our sample is quite large. The nursing homes in which the study was conducted, on the basis of Italian law, admit mainly elderly subjects, but above all, subjects with a high degree of frailty. Only 17 subjects less than 65 years and 14 less than 60 years were enrolled in our study. The elimination of subjects under the age of 65 did not modify the results of the study.

The study has a number of strengths. First, a nutritional evaluation was performed on all the nursing homes’ residents, thus avoiding selection biases. In particular, cognitive impairment, disability, and comorbidity did not impact recruitment, allowing a greater representative sample. Second, a multidimensional evaluation was performed and allowed for a more precise definition of the individual role played by different risk factors on nutritional status.

## 5. Conclusions

Age and physical and mental health were found to be significantly associated with poor nutritional status. This result suggests to assess nutritional status and investigate the presence of risk factors associated with malnutrition in order to achieve effective prevention and plan a better intervention strategy. In particular, in future studies, the quality of assistance in nursing homes should be considered. For many years, different aspects of nutritional care have been highlighted as important determinants of nutritional status in hospitals and nursing homes (https://www.nutritionday.org/cms/upload/pdf/11.resolution/Resolution_of_the_Council_of_Europe.pdf; accessed on 25 August 2020). These parameters are not surveyed in studies concerning malnutrition, even if their consideration and correction could contribute to significantly improve the nutrition status of residents in nursing homes.

## Figures and Tables

**Table 1:** Patient clinical, functional, and nutritional characteristics.

		Total Sample	Males	Females	*p*
*N*		246	82	164	
	Age (years)	80.5 ± 10.3	76.5 ± 11.0	82.5 ± 9.3	<0.001
CIRS scale	Number High Comorbidity Index (>4) (%)	25 (10.2)	10 (12.4)	15 (9.2)	0.436
	Number High Severity Index (>1.5) (%)	113 (46.1)	34 (42.0)	79 (48.2)	0.360
ADLs	Median Score (IQR)	8 (4, 10)	9 (6, 11)	7 (4, 10)	0.001
Physical Performance-SPPB battery	Median score (IQR)	1 (0, 4)	2 (0, 5)	0 (0, 3)	0.001
	Reduced physical performance (score ≤ 4) (%)	200 (81.3)	61 (74.4)	139 (84.8)	0.090
MMSE	Median score (IQR)	16 (13, 19)	17 (14, 20)	16 (12.5, 19)	0.103
	Number Cognitive Status Impaired (score ≤ 18) (%)	163 (66.5)	48 (59.3)	115 (70.1)	0.090
Depression (GDS scale)	Mean score (SD)	6.9 (3.3)	6.8 (3.4)	6.9 (3.3)	0.816
	Number Depression (score > 10/15) (%)	63 (25.7)	18 (22.2)	45 (27.4)	0.628
Nutritional Status (M-MNA)	Mean score (SD)	13.1 (3.1)	13.5 (2.8)	12.9 (3.2)	0.193
	Number malnourished (score < 17) (%)	48 (19.5)	12 (14.6)	36 (22.0)	0.314
	Number at-risk of malnutrition (score 17–23.5) (%)	94 (38.2)	31 (37.8)	63 (38.4)	
	Number normal nutritional status (score ≥ 24) (%)	104 (42.3)	39 (47.6)	65 (39.6)	

CIRS scale: Cumulative Illness Rating Scale, ADL: Activities of Daily Living, IQR: interquartile range, MMSE: Mini Mental State Examination; M-MNA: Modified Mini Nutritional Assessment, GDS: Geriatric Depression Scale.

**Table 2:** Association between each factor and reduced M-MNA total score (≤14).

Variable	Adjusted R-Squared	Beta Coefficient (SE) *	95%CI	*p*-Value
Disability	0.266	0.463 (0.052)	0.359 to 0.566	<0.001
Health Status-Index of Severity (CIRS severity)	0.034	−1.085 (0.847)	−2.754 to 0.584	0.202
Health Status-Multi-morbidity Index (CIRS comorbidity)	0.040	−0.218 (0.127)	−0.467 to −0.032	0.087
Physical Performance (SPPB)	0.101	0.327 (0.082)	0.166 to 0.489	<0.001
Depression (GDS)	0.096	−0.237 (0.057)	−0.349 to −0.124	<0.001
Cognitive Function (MMSE)	0.074	0.135 (0.041)	0.054 to 0.216	*p* = 0.001

* All models were controlled for age and gender. Legend. CIRS: Cumulative Illness Rating Scale; SPPB: Short Physical Performance Battery; GDS: Geriatric Depression Scale; MMSE: Mini Mental State Examination; M-MNA: Modified Mini Nutritional Assessment.
